# Impact of the initiation of isCGM soon after type 1 diabetes mellitus diagnosis in adults on glycemic indices and fear of hypoglycemia: a randomized controlled trial

**DOI:** 10.3389/fendo.2024.1503891

**Published:** 2025-01-09

**Authors:** Jerzy Hohendorff, Agata Grzelka-Wozniak, Marta Wrobel, Michal Kania, Lidia Lapinska, Dominika Rokicka, Dorota Stoltny, Irina Kowalska, Krzysztof Strojek, Dorota Zozulinska-Ziolkiewicz, Maciej T. Malecki

**Affiliations:** ^1^ Department of Metabolic Diseases, Jagiellonian University Medical College, Krakow, Poland; ^2^ Internal Medicine, Metabolic Diseases and Diabetology Clinical Department, University Hospital in Krakow, Krakow, Poland; ^3^ Department of Internal Medicine and Diabetology, Poznan University of Medical Sciences, Poznan, Poland; ^4^ Department of Internal Medicine, Diabetology and Cardiometabolic Disorders, Faculty of Medical Sciences Zabrze, Medical University of Silesia, Katowice, Poland; ^5^ Department of Internal Medicine and Metabolic Diseases, Medical University of Bialystok, Bialystok, Poland

**Keywords:** isCGM, fear of hypoglycemia, TIR, TBR, type 1 diabetes

## Abstract

**Background:**

Continuous glucose monitoring (CGM) improves glycemic control and quality of life. Data on glycemic indices and fear of hypoglycemia (FoH) in newly diagnosed T1DM patients are limited.

**Aim:**

To assess the impact of initiating intermittently scanned CGM (isCGM) within 1–6 months of diagnosis on glycemic control and FoH in adults with T1DM.

**Subjects and methods:**

After wearing a blinded sensor for 14 days, participants were randomized (1:1) to either isCGM (intervention) or self-monitoring blood glucose (SMBG) with glucometers and blinded CGM (control). Primary outcomes were changes in time below 70 mg/dl (TB70) and FoH, assessed in the Hypoglycemia Fear Survey (HFS). Main secondary outcomes included changes in mean glucose and time in range (TIR) from baseline to 4 weeks after randomization.

**Results:**

The full analysis set included 23 patients (12 from the intervention group and 11 from the control group), aged 25.6 ± 5.1 years (14 men, 9 women). All participants were on multiple daily insulin injections. TB70 changed from 2.42% to 2.25% in the intervention, and from 2.81% to 1.82% in the control group, and the between-therapy difference of 0.83% was insignificant. No difference between intervention and control groups in change in HFS-worry and HFS-behavior subscales between baseline and after 4 weeks was found (−1.6 ± 3.2 and 1.0 ± 2.2, respectively). The mean glucose levels changed from 7.03 mmol/l to 6.73 mmol/l and from 7.07 mmol/l to 7.43 mmol/l, in the intervention and control groups, respectively, which resulted in a between-therapy significant glucose difference of −0.66 mmol/l. The mean TIR changed from 88.0% to 90.0% in the intervention group and from 85.2 to 84.1% in the control group—the between-therapy difference was insignificant (3,1%). The study ended early due to CGM reimbursement policy changes, after which most patients eligible for the study could have isCGM reimbursed.

**Conclusions:**

In newly diagnosed T1DM adults, TIR is high and hypoglycemia risk is low. The study group was small; however, the data suggest that the use of isCGM soon after T1DM diagnosis could result in mean glucose decrease, but not in change in TB70 and FoH.

## Introduction

Use of continuous glucose monitoring (CGM) systems has become the standard of glucose control in most patients with type 1 diabetes (T1DM) (1). It is recommended by the American Diabetes Association (ADA) to initiate CGM early in T1DM, even at time of diagnosis (2). There are two types of CGM—intermittently scanned CGM (isCGM) and real-time CGM (rtCGM) (3). It is well proven that CGM systems increase time in range (TIR), reduce the number and duration of hypoglycemic episodes, reduce time spent in hyperglycemia, and improve the quality of life (QoL) (4–6). QoL in people with T1DM is decreased due to a need of performing insulin injections, frequent glucose measurements, and carbohydrate intake counting needed to prevent development of chronic complications (7, 8). The first few months after the diagnosis can be particularly difficult as the newly diagnosed patients need to adapt to the reality of a chronic and challenging disease. The diagnosis of T1DM is usually followed by a transient improvement in blood glucose levels and a drop in insulin requirement, which sometimes meets the criteria of a so-called “partial clinical remission” (PCR) (9–11). The phenomenon of PCR occurs in approximately 25%–60% of adult T1DM patients (11–13). In some rare cases of a total diabetes remission, insulin treatment can be even temporarily interrupted (14). PCR typically starts shortly after diagnosis, usually no later than by the end of the 12th month, and lasts for several months (12, 15). During PCR, due to a substantial drop in the insulin requirements and a challenge to adjust its dose, the higher risk of mild hypoglycemia is observed (16). While limited data on severe hypoglycemia episodes in adults with T1DM during this period were published, such patients were seen in the authors’ clinical practice. Possible severe hypoglycemic events may cause dramatic psychological trauma and affect the future treatment efficacy and quality of life for years to come in this population. Clinical data on the initial period of T1DM in adults regarding this initial drop in insulin requirement, particularly in terms of hypoglycemia, fear of hypoglycemic episodes, and the quality of life, are limited, especially on an effect of isCGM on hypoglycemic episodes and psychological well-being of newly diagnosed patients with T1DM.

The aim of this study was to evaluate the impact of the isCGM system on glycemic control and assess the fear of hypoglycemic episodes as well as quality of life in young adults with newly diagnosed T1DM.

## Materials and methods

This prospective, randomized, non-masked study was conducted in four academic centers in Poland—Krakow, Poznan, Zabrze, and Bialystok (ClinicalTrials.gov reg. no. NCT06414824). Patients were eligible for the study if aged 18–35 years, newly diagnosed T1DM (1–6 months), treated with multiple daily injections of insulin (MDI), and in the investigator’s opinion technically capable of using isCGM. Patients were not included if they had used any CGMS or were on pump therapy, were pregnant or were planning pregnancy or breast feeding, were participating in another clinical trial that could affect glucose measurements or glucose management, had known allergy to medical adhesives, had severe end-organ damage (kidney, liver), and were diagnosed with psychiatric disorders.

After 14 days of wearing a blinded sensor, participants were randomly assigned by the central interactive web response system in a ratio 1:1 to isCGM (intervention group) or to self-monitoring of blood glucose (SMBG) with glucometers and blinded CGM (control group) using Randomizer for Clinical Trials developed by the Institute for Medical Informatics, Statistics and Documentation, Medical University of Graz (www.randomizer.at). The big stick randomization method was used, with equal probabilities assigned to each group, until a prespecified maximum tolerated imbalance of 3 was reached. Neither site nor sex was used as a factor for subgroup stratification. Participants needed to achieve a minimum of 70% wear time of the blinded glucose sensor prior to being randomized. Neither participants nor investigators were masked, which is a common feature of similar studies (4). The primary outcomes were change in time below 70 mg/dl (TB70) and fear of hypoglycemia assessed (FoH) in Hypoglycemia Fear Survey (HFS) from baseline to 4 weeks after randomization between study groups, and secondary outcomes were change in CGM-derived metrics: mean glucose, glucose management indicator (GMI), time in range (TIR), time above 180 mg/dl (TA180), time above 250 mg/dl (TA250), time below 54 mg/dl (TB54), coefficient of variation (CV) from baseline to 4 weeks after randomization, and a difference in the Diabetes Distress Scale (DDS) and in the Diabetes Treatment Satisfaction Questionnaire (DTSQ) at the end of study between study groups (17–20). The DDS questionnaire contains 17 items across four fields: emotional burden (five items), physician distress (four items), regimen distress (five items), and interpersonal distress (three items). Each item is scored on a 6-point scale: forms 1 (“not a problem”) to 6 (“very significant problem”). The average score of <2.0 is no or little distress, 2.0–3.0 reflects moderate distress, and >3.0 high distress. A score of >2.0 is considered as clinically significant distress. DTSQs (status) contains eight items scored on a 7-point scale ranging from 0 (“very dissatisfied,” “very inconvenient”) to 6 (“very satisfied,” “very convenient”) and indicates patients’ satisfaction at baseline. DTSQc (change) contains seven items scored on a 7-point scale ranging from −3 to 3. High scores indicate much more satisfied, convenient, or likely to recommend, whereas low scores indicate dissatisfied, inconvenient, and unlikely to recommend new therapy. HFS contains two subscales: behavior (15 items) and worry (17 items). All items are rated on a 5-point scale (0 = “never” to 4 = “almost always”). Higher scores indicate higher fear of hypoglycemia.

In a large study on isCGM in adults with well-controlled type 1 diabetes, the baseline TB70 was 14.2% ± 10%, and the FoH-adjusted mean score was approximately 30 ± 5 (4). Our prediction was that the study population would likely have lower TB70, which we estimated at 8% ± 5%. One of the main reasons we estimated a lower TB70 is that the current isCGM system has a lower mean absolute relative difference. To detect a difference to a mean improvement of 40% TB70 (to the target TB70 of 5% set by the International Consensus (17)), at 80% power and α of 0.05, we would need 45 participants in each arm. Similarly, assuming a 10% improvement in HFS, 45 participants in each arm would also be required.

The study was approved by the local bioethics committee. Participants gave written informed consent prior to entering the study. Statistical analyses were performed using IBM SPSS Statistics (version 29). Final analyses were performed using per-protocol treatment population. A significance level of p=0.05 was used in the analyses. Quantitative variables were checked for normal distribution. Parametric t test or non-parametric U tests were performed, where applicable, to describe clinical characteristics and differences between the study groups. For nominal variables, the Fisher’s exact test was used.

## Results

**Figure 1:**
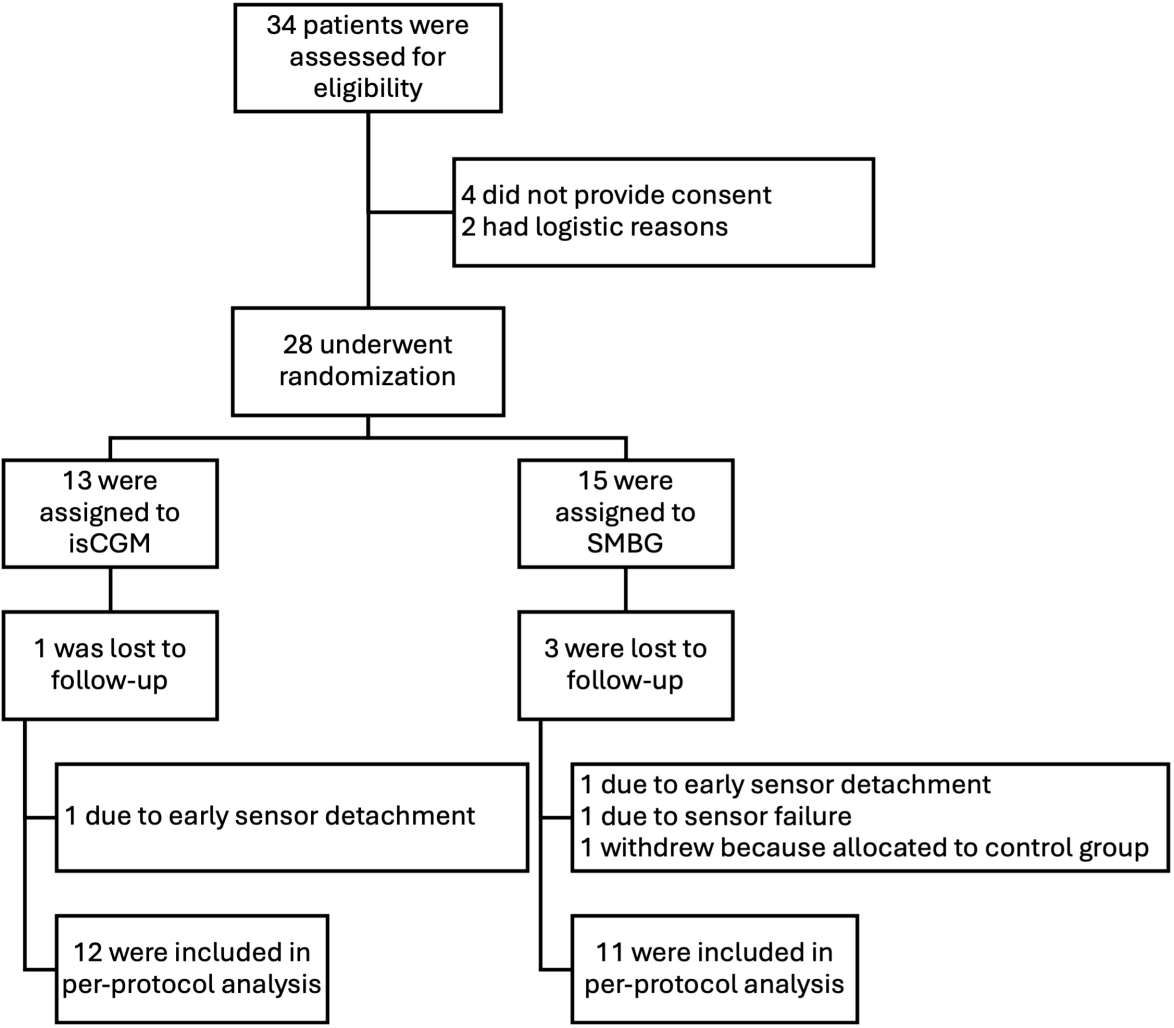
CONSORT flow diagram.

There were 28 patients enrolled between May 2022 and December 2023. After completing the baseline 14-day phase of wearing a blinded sensor, the patients were randomly assigned to the intervention group (n=13) and control group (n=15). The full analysis set included 23 patients (12 from the intervention group and 11 from the control group). One patient withdrew consent to participate in the study, and four patients were excluded from analysis due to sensor failure or early sensor detachment (**Figure 1**).

The study was terminated early due to changes in reimbursement policy in Poland, as during the study groups of patients eligible for reimbursement were significantly expanded; particularly all patients who would be eligible for the study could have isCGM reimbursed. Moreover, the next generation of studied device, Libre 2, formally still isCGMS, but having features of rtCGMS, was introduced on the Polish market (21).

There was no difference between the intervention and control groups in terms of sex (men: 9 in 12 vs. 5 in 11) and age (26.3 ± 5.2 vs. 24.8 ± 5.2 years). At diabetes diagnosis, HbA1c% was similar between intervention and control groups (12.1 ± 2.2% vs. 14.2 ± 3.1%). At baseline, the mean BMI was lower in the intervention than control group (19.3 ± 4.7 vs. 23.9 ± 3.8 kg/m^2^). The mean daily dose of insulin did not differ between intervention and control groups (0.38 ± 0.22 vs. 0.44 ± 0.20 IU/kg), and the number of patients with daily insulin requirement less than 0.5 IU/kg was 7 in each group (**Table 1**).

**Table 1:** Subjects’ characteristics.

	Intervention group (isCGM) N=12	Control group (SMBG) N=11	p
Age (years)	26.3 ± 5.2	24.8 ± 5.2	0.49
Sex (female/male)	3/9	6/5	0.21
BMI (kg/m^2)^	19.3 ± 4.7	23.9 ± 3.8	0.02
HbA1c at diabetes diagnosis (%)	12.1 ± 2.2	14.2 ± 3.1	0.11
Daily dose of insulin at baseline (IU/day)	0.38 ± 0.22	0.44 ± 0.20	0.52
Number of subjects with daily dose of insulin (IU/day) >0.5/0.5-0.3/<0.3	5/2/5	4/4/3	0.67

Data shown as mean ± SD. Differences in quantitative variables were analyzed using t-test, whereas differences in qualitative variables were assessed using Fisher’s exact test.

**Figure 2:**
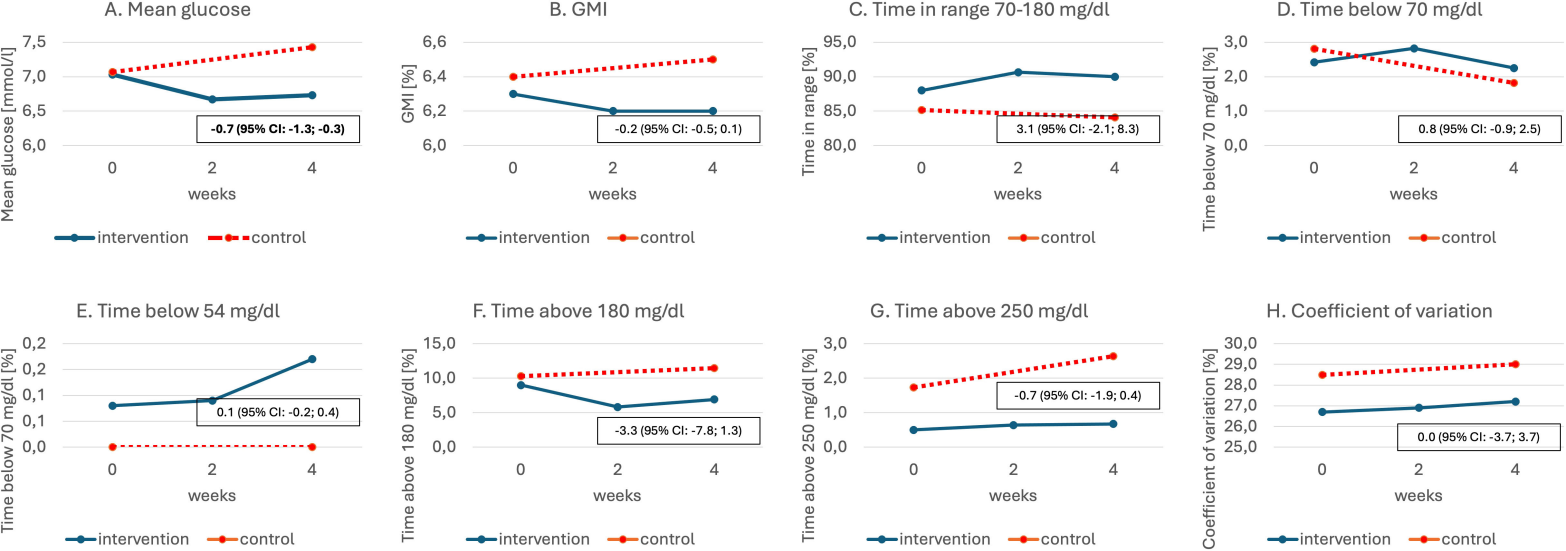
Mean glycemic indices among the study groups by week of the study: **(A)** Mean glucose; **(B)** GMI; **(C)** Time in range 70-180 mg/dl; **(D)** Time below 70 mg/dl; **(E)** Time below 54 mg/dl; **(F)** Time above 180 mg/dl; **(G)** Time above 250 mg/dl; **(H)** Coefficient of variation. Number in boxes indicate differences in mean change between therapies (intervention vs control) after 4 weeks. Bold values denote statistical significance.

The mean time spent in hypoglycemia <70 mg/dl changed from 2.42% to 2.25% in the intervention group, and from 2.81% to 1.82% in the control group, and the between-therapy difference of 0.83% was insignificant (**Figure 2**; **Table 2**). No significant difference between intervention and control groups in change in HFS-worry and HFS-behavior subscales between baseline and after 4 weeks was found (−1.6 ± 3.2 and 1.0 ± 2.2, respectively) (**Table 3**). However, due to a smaller than planned sample size, the analysis was underpowered.

**Table 2:** CGM-delivered data—baseline and 4 weeks after randomization.

	Baseline			End of study				
	Intervention group (isCGM) N=12	Control group (SMBG) N=11	p	Intervention group (isCGM) N=12	Control group (SMBG) N=11	Difference between therapies	95% CI	p
Mean glucose [mmol/l]	7.03±0.90	7.07±1.43	0.937	6.73±0.83	7.43±1.47	−0.66±0.30	−1.3, −0.3	0.041
GMI [%]	6.3±0.4	6.4±0.6	0.823	6.2±0.4	6.5±0.6	−0.2±0.1	−0.5, 0.1	0.102
TB54 [%]	0.08±0.29	0.00±0.00	0.350	0.17±0.39	0.00±0.00	0.08±0.16	−0.2, 0.4	0.598
TB70 [%]	2.42±2.71	2.81±2.32	0.708	2.25±2.05	1.82±1.60	0.83±0.81	−0.9, 2.5	0.317
TIR [%]	88.00±8.44	85.18±15.77	0.594	90.00±7.42	84.09±16.62	3.09±2.52	−2.1, 8.3	0.233
TA180[%]	9.00±7.82	10.27±11.88	0.762	6.92±6.52	11.45±12.23	−3.27±2.20	−7.8, 1.3	0.152
TA250 [%]	0.50±1.00	1.73±4.45	0.362	0.67±0.89	2.64±5.03	-0.74±0.55	−1.9, 0.4	0.192
CV [%]	26.7±5.6	28.5±3.9	0.381	27.2±5.8	29.0±4.4	−0.02±1.81	−3.7, 3.7	0.993

Data shown as mean ± SD. Differences between study groups were analyzed using t-test, but TBR using the U-test.

**Table 3:** Scores from DTSQ, DDS, and HFS questionnaires—baseline and 4 weeks after randomization.

	Baseline			End of study				
	Intervention group (isCGM) N=12	Control group (SMBG) N=11	p	Intervention group (isCGM) N=12	Control group (SMBG) N=11	Difference between therapies	95% CI	p
HFS-W	17.3±7.7	20.5±10.0	0.409	12.3±5.3	17.0±10.4	−1.6±3.2	−5.0, 8.2	0.614
HFS-B	18.5±6.1	18.0±8.0	0.867	14.3±5.1	12.7±7.3	1.0±2.2	−5.5, 3.5	0.643
DDS total	46.8±16.0	48.49±21.60	0.800	45.4±17.6	44.0±17.8	3.4±7.0	−18.1, 11.2	0.627
DDS emotional	15.9±6.1	16.3±6.3	0.887	14.9±6.1	13.6±4.9	1.7±2.1	−2.6, 6.0	0.419
DDS physician	11.1±5.1	12.6±6.9	0.575	9.9±5.0	10.9±6.3	0.5±2.7	−5.2, 6.3	0.852
DDS regimen	12.3±4.7	13.7±6.3	0.563	13.4±4.6	12.8±4.3	2.0±2.1	−2.4, 6.4	0.357
DDS inter-personal	7.5±3.2	6.3±3.9	0.429	7.2±3.3	6.7±3.9	−0.8±1.3	−3.4, 1.9	0.550
DTSQs	31.6±4.2	29.0±6.4	0.261	N/A	N/A	N/A	N/A	N/A
DTSQc	N/A	N/A	N/A	12.1±4.6	10.5±4.3	1.6±1.9	−5.5, 2.2	0.389

DDS, diabetes distress scale; DTSQs/c, diabetes treatment satisfaction questionnaire: status/change; HFS W/B, hypoglycemia fear survey: worry subscale/behavior subscale. Differences between study groups were analyzed using t-test (HFS), and U-test (DDS, DTSQ).

The mean glucose level changed from 7.03 mmol/l to 6.73 mmol/l in the intervention group and from 7.07 mmol/l to 7.43 mmol/l in the control group, which resulted in a between-therapy significant difference of −0.66 mmol/l. No statistically significant difference was found in between-group change in GMI, TA180, TA250, TB54, and CV. Detailed data on glycemic indices are shown in **Figure 2** and **Table 2**. Patient satisfaction with treatment did not change significantly when compared intervention with control (**Table 3**). Similarly, no difference in change in diabetes distress was found (**Table 3**).

No event of severe hypoglycemia (requiring hospitalization or third-party intervention) and no event of diabetic ketoacidosis were reported during the study.

## Discussion

In this multicenter randomized controlled trial, we have examined the impact of soon-after T1DM diagnosis introduction of isCGM in adults on glycemic indices, diabetes treatment satisfaction, diabetes distress, and FOH.

First, we have shown that within first months after T1DM diagnosis, patients maintain good glycemic control, as baseline TIR was very high. This likely depends, at least partially, on PCR of T1DM, during which daily insulin requirement drops. Different criteria are proposed to diagnose PCR; most are based on insulin requirements, eq. daily dose of insulin of <0.5 IU/kg or <0.3 IU/kg, or an insulin dose-adjusted A1C (A1c [%]*4*DDI [IU/kg] <9.0) (9, 22). In our study, most patients’ daily insulin requirement was less than 0.5 IU/kg. Analyzed glycemic indices seem to be much better if compared with published data of the general T1DM population of longer diabetes duration, as in the presented study the mean baseline TIR was close to 90% and in different T1DM populations varies widely from 50% to 85% (23–26). In recently published studies, where glycemic control was analyzed in children and adolescents with newly diagnosed T1DM, TIR was ca. 70% (27, 28). To the best of our knowledge, no similar study in adults with newly diagnosed T1DM was published within the last years.

Second, we have shown that even in patients with so high a percentage of TIR, introducing CGM significantly decreased mean glucose. While TIR and GMI appeared to improve, the changes were not statistically significant. This lack of significance is likely due to underpowered analyses resulting from the early termination of the study and a lower than planned number of subjects examined. Use of CGM resulted in improvement in mean glucose, GMI, TIR, time above range (TAR), and time below range (TBR), which was well documented in many studies so far. However, in none of these studies, baseline TIR was close to 90% (4, 27–30).

Next, in our study, no change in TBR and patients’ FoH was observed. However, findings related to TBR and FOH should be considered preliminary due to the early termination of the study and reduced statistical power. Such change could likely be observed in patients with high baseline FoH, such as those with a history of severe hypoglycemic events. Moreover, in a longer study, with more hypoglycemic events, the effect of isCGM could be more clearly demonstrated. Data from some observational studies suggest lower FoH in patients using isCGM, especially in subjects with impaired awareness of hypoglycemia (31–34). Worth noting is that, in our previous study, we have shown that lower FoH is observed in patients performing more daily scans compared with those who perform fewer (23). Results of RCT provided divergent data. A large RCT investigating isCGM in adults with well-controlled T1DM, performed in 2016, did not show a positive effect of isCGM on hypoglycemia worry and hypoglycemia behavior (4, 34, 35). However, in that experiment, a positive effect of isCGM on treatment satisfaction score was seen (4). This finding is confirmed by other studies in which isCGM was tested versus SMBG (31, 33). In our study, we have not shown improvement in results of DDS and DTSQ.

We must acknowledge that our study has some limitations. First, we terminated the study early; thus, sample size is smaller than it was planned, so some expected effects of isCGM use might not be seen. Among eligible patients, some percentage did not provide consent, primarily because they did not want to be assigned to the control group. Additionally, this was the reason one patient withdrew consent. Moreover, the study was rather short-term, and some effects could need a longer study duration to be proofed. The strength of the study is its randomized nature in a specific group of patients with short-term duration of diabetes. However, despite randomization, an imbalance between the study groups was found in terms of BMI, which could lead to bias in the estimates of the treatment effects, in particular the observed difference in change in mean glucose.

The results of our study should be interpreted in the context of the long-term effects of isCGM, as its use leads to sustained improvements in glycemic control (30, 36). Use of isCGM improves not only glycemic indices but other important clinical outcomes as well. In real-world settings of FUTURE study, use of isCGM was associated with fewer hospitalizations due to hypoglycemia and/or diabetic ketoacidosis and less workplace absenteeism (31).

Accessibility and reimbursement policies of CGM systems differ among countries limiting common use of CGM worldwide. Thus, results of the present study, particularly improvement in mean glucose, support recommendation on early use of CGM, even from a day of diagnosis.

Since the use of CGM is considered the standard for glucose monitoring in T1DM and early use is recommended by the ADA, future research could focus on which features and what settings of CGM would provide most benefits to a specific group of patients.

## Conclusions

In adults, the first months after T1DM diagnosis are associated with good glycemic control, high TIR, and low hypoglycemia risk. Early initiation of isCGM use soon after T1DM diagnosis was not associated with either TB70 or FoH reduction; however, the mean glucose level was decreased. Additionally, a trend to further increase TIR was seen. Given the early termination of the study and the small sample size, the results of the study should be considered as preliminary.

## Data Availability

The raw data supporting the conclusions of this article will be made available by the authors, without undue reservation.
